# CO_2_ Self-Sufficiency in Renewable Methanol Production from Biomethane and Biohydrogen: Techno-Economic Assessment for Spain

**DOI:** 10.3390/bioengineering13101183

**Published:** 2026-10-11

**Authors:** Arantza Rascón, José Ignacio Linares, Ana María Santos-Montes, Eva Arenas

**Affiliations:** 1 Rafael Mariño Chair in New Energy Technologies, Comillas Pontifical University, Alberto Aguilera 25, 28015 Madrid, Spain; 201804003@alu.comillas.edu (A.R.); earenas@comillas.edu (E.A.); 2 Repsol Foundation Chair in Energy Transition, Comillas Pontifical University, Alberto Aguilera 25, 28015 Madrid, Spain; asantos@comillas.edu; 3 Institute for Research in Technology, Comillas Pontifical University, Santa Cruz de Marcenado 26, 28015 Madrid, Spain

**Keywords:** biomethanol, biomethane, biohydrogen, biogenic CO_2_

## Abstract

Renewable methanol is commonly proposed either as biomethanol from biomass or biomethane reforming, or as e-methanol from CO_2_ hydrogenation using electrolytic hydrogen. This work proposes a third concept: CO_2_-self-sufficient biomethanol from biomethane, where biohydrogen and biogenic CO_2_ are internally produced through biomethane reforming with carbon capture and utilisation. Four process routes are assessed for Spain, combining conventional CO/H_2_ methanol synthesis and CO_2_/H_2_ hydrogenation. The proposed routes use biohydrogen rather than electrolytic hydrogen. In route 1 (baseline case), fossil methane and CO_2_ in conventional methanol production are replaced by renewable alternatives. Route 2 produces hydrogen and CO_2_ through steam biomethane reforming with carbon capture and utilisation, generating a CO_2_ surplus. Route 3 uses a fraction of biomethane to produce CO_2_ and biohydrogen, supplying the CO_2_ required by route 1 and yielding excess hydrogen. Finally, route 4 integrates routes 1 and 2, using the CO_2_ surplus from route 2 as feedstock for route 1. Routes 1 and 4 produce exclusively methanol and achieve the highest yields (108.6 t and 102.7 t per GWh-HHV of biomethane, respectively). All routes except route 1 are CO_2_ self-sufficient. A detailed techno-economic assessment of routes 1 and 4 gives methanol costs of 403–817 €/t and 496–933 €/t, respectively.

## 1. Introduction

### 1.1. Current Production of Methanol

Methanol (MeOH) is a key building block in the chemical industry. Around 70% of global production is used to manufacture essential chemicals, including olefins, formaldehyde, acetic acid, and methyl tert-butyl ether (MTBE), while the remaining 30% is mainly used in road transport [1]. In 2023, global methanol production reached 110 Mt [2], with China as the largest producer. Production is expected to grow further, to about 500 Mt by 2050 [2]. These projections underline the role of methanol not only as an essential feedstock in the chemical industry but also as an emerging fuel, particularly in sectors such as maritime transport.

Currently, most methanol is produced from natural gas and coal via the synthesis gas (CO/H_2_) route [1]. For natural gas, methane is reformed to produce synthesis gas; for coal, synthesis gas is obtained through gasification. These fossil-based methanol routes lead to significant CO_2_ emissions, ranging from 0.5 kg CO_2_/kg MeOH for natural gas to 2.6–3.8 kg CO_2_/kg MeOH for coal [1]. Life-cycle emissions from current methanol production and use are estimated at around 0.3 Gt CO_2_ per year. If production grows as projected and demand is met using fossil-based methanol, annual emissions could increase to 1.5 Gt CO_2_ per year by 2050 [3].

### 1.2. Renewable Methanol

There are two main categories of low-emission methanol: biomethanol and e-methanol. Biomethanol is obtained from biological feedstocks, such as solid biomass (using a process similar to coal gasification) or biomethane (with a treatment similar to natural gas reforming). E-methanol, on the other hand, is produced by catalytic CO_2_ hydrogenation (CO_2_/H_2_) using CO_2_ captured from biogenic sources, from unabated industrial emissions, or directly from air, together with renewable hydrogen obtained via electrolysis powered by renewable electricity [1].

The costs of biomethanol and e-methanol remain uncertain due to the current low level of deployment, but they are primarily driven by feedstock prices, investment costs, and process efficiency. The main barrier to the adoption of low-emission methanol is its high cost compared with fossil methanol. The production cost of fossil (conventional) methanol is approximately 250 $/t (Currency has been left as in the original reference (dollars or euros). When conversion has been necessary (i.e., in the economic model), an equivalence of 1 $ = 0.85 € has been applied), although it varies with raw material prices, production methods, plant size, and other factors [3]. In contrast, the cost of e-methanol is highly sensitive to the prices of CO_2_ and H_2_: using biogenic CO_2_, current costs lie in the range 800–1600 $/t, while with direct air capture (DAC) they increase to 1200–2400 $/t [1]. For biomethanol produced from biomass by gasification, costs range between 450 and 750 $/t. When biomethane is used, its cost depends strongly on the feedstock, with reported values in the range 410–425 $/t [1].

The increasing demand for low-carbon methanol has prompted a substantial rise in research on new synthesis routes. Numerous recent studies have investigated different feedstocks—both fossil and renewable—and various process configurations aimed at reducing environmental impacts. A large body of work evaluates the levelised cost of methanol (LCOM) and its main cost components across a range of technologies and raw materials and can be summarised as follows.

### 1.3. Methanol from Syngas

Some studies examine routes that utilise syngas from reforming technologies using natural gas as feedstock. For instance, Ren et al. [4] obtain cost-effective methanol (around 100 $/t) by integrating CO_2_ captured from power plant flue gases into the synthesis process, thus achieving a significant reduction in emissions. Lim et al. [5] incorporate renewable hydrogen from different electrolyser types into a natural gas tri-reforming process; the resulting cost is higher and strongly dependent on the electrolyser technology. Arnaiz del Pozo et al. [6] compare several natural gas reforming routes with an alternative process based on hydrogenating captured CO_2_ using electrolytic hydrogen and project e-methanol costs to 2050 to assess their potential future competitiveness against conventional processes.

Other studies evaluate biomethanol production via syngas routes using reforming processes. Rinaldi et al. [7] focus on converting biogas from municipal solid waste (MSW) and explore three configurations for CO_2_ separation: before reforming, after reforming, and during post-synthesis purification. The configuration that separates CO_2_ prior to reforming is identified as the most favourable, with the associated costs reported by the authors. In contrast, Kgwedi et al. [8] investigate biomethanol from glycerol, a by-product of biodiesel synthesis, finding production costs in the range of 850–981 €/t.

A further group of studies addresses biomethanol production via biomass gasification. De Fournas and Wei [9] analyse two California biorefineries producing marine fuel and compare a configuration with carbon capture and storage (CCS) against a hybrid configuration with proton exchange membrane electrolysis, including the role of carbon credits. Lombardelli et al. [10] compare five smaller-scale European configurations that differ in gasification pressure and in their use of CO_2_ capture technologies or green hydrogen, identifying options that minimise methanol cost and net CO_2_ emissions. Wang et al. [11] investigate the feasibility of producing biomethanol from corn straw in China. Tariq et al. [12] propose an integrated process combining biomass gasification with hydrogenation of CO_2_ captured from synthesis gas using renewable electrolytic hydrogen; their life-cycle assessment shows nearly complete CO_2_ emission abatement. Reported costs for green methanol (biomethanol+ e-methanol) range from 413 to 813 €/t, depending mainly on biomass prices, electricity costs, and investment assumptions.

Niu et al. [13] compare three pathways for methanol production—natural gas steam reforming, biomass gasification, and steam reforming of biogas derived from agricultural residues—using energy, exergy, economic, and environmental indicators. Their results indicate that natural gas reforming is currently the most cost-effective option, followed by biogas reforming and biomass gasification, while the latter two routes show greater long-term potential as the costs of renewable feedstocks decrease. Chen et al. [14] compare two methanol production pathways from distillers’ grains: (i) direct gasification and (ii) an integrated approach combining anaerobic digestion with digestate gasification. In the integrated route, both biogas and process-derived gas are reformed into synthesis gas for methanol production. Although this integrated approach improves conversion efficiency, it also increases process complexity compared to direct gasification. The reported production costs are 264.73 $/t for direct gasification and 292.59 $/t for the integrated route. Marques da Cunha et al. [15] propose a system that uses a Carnot battery to produce methanol from biogas via bi- and tri-reforming processes, using renewable electricity to meet thermal and electrical demands. Their model indicates that production costs may increase from a baseline of 170–260 $/t to 300–600 $/t, while achieving CO_2_ emission reductions of up to 84%, with tri-reforming identified as the most efficient method.

In a recent study, Enayati et al. [16] evaluate the yields and exergy efficiencies of two innovative methods for converting biogas into biomethanol. The first method involves directly steam reforming raw biogas, followed by methanol synthesis, whereas the second upgrades biogas to biomethane and then reforms it to methanol. The findings indicate that the first pathway maximises methanol production, while the second offers better environmental performance by achieving greater emission reductions. However, the study does not include an economic assessment. The reviewed studies on methanol synthesis via the syngas route are summarised in Table 1.

### 1.4. E-Methanol

Recent literature shows strong interest in e-methanol via CO_2_ hydrogenation using electrolytic green hydrogen. Among the reviewed works, this is the most widely studied low-carbon methanol technology, although LCOM varies widely (520–4510 €/t). Several studies rely on CO_2_ captured from different thermoelectric power plants: in Battaglia et al. [17] CO_2_ is captured from a coal-fired power plant, in Sollai et al. [18] it is captured from a fossil-fuel thermal plant, in Wei et al. [19] from a conventional power plant, and in Han et al. [20] from an oxy-fuel combined-cycle facility. In all cases, hydrogen is produced from renewable electricity, including hydropower, surplus renewable energy, or solar– wind hybrid systems. In Nizami et al. [21] and Gu et al. [22], CO_2_ comes from industrial processes: in Nizami’s work, from natural gas field processing, with PV-grid and PV-battery power supply options compared. The findings indicate that while PV-grid is currently the more affordable option, anticipated future cost reductions in photovoltaics, battery storage, and electrolysis could enhance the competitiveness of PV-battery systems, potentially achieving near-zero emissions. In Gu’s study, CO_2_ is derived from coal-based methanol production. Cormos [23] analyses different CO_2_ sources and also considers synthetic natural gas production. On a larger spatial scale, Rodríguez-Pastor [24] performs a techno-economic and geographic assessment across Europe, identifying over 3000 potential “green methanol valleys” that could couple CO_2_ from energy-intensive industries with green hydrogen under future cost reductions for electrolysers and CO_2_ capture.

Additionally, Siskos et al. [25] provide an integrated techno-economic and environmental assessment of a carbon capture and utilisation (CCU) plant converting CO_2_ from a cement facility into methanol, identifying green hydrogen as the main economic hotspot. Gelten et al. [26] evaluate a decentralised system in which CO_2_ released during biogas upgrading is converted back into methanol using electrolytic hydrogen and find that renewable hydrogen operational costs dominate capital expenditures. Bos et al. [27] examine direct air capture as a CO_2_ source combined with wind-based hydrogen. Wang et al. [28] describe an integrated off-grid system that combines wind and solar power, diesel backup, and pumped storage for hydrogen production. This configuration enables the conversion of CO_2_ into methanol, thereby enhancing both process stability and economic viability. A techno-economic analysis reveals that, if the price of renewable electricity drops to 0.012 €/kWh (cost reported in CNY in the reference; for comparison, the equivalent value in € was calculated using an average exchange rate of 1 CNY = 0.12 €) [29], the methanol production cost could decrease to 312 €/t, making it competitive with coal-based methods. Zhou et al. [30] evaluate a comprehensive system for converting municipal solid waste into green methanol. This process includes waste incineration, CO_2_ capture, electrolysis powered by renewable electricity, methanol synthesis, and waste heat recovery through an organic Rankine cycle. Notably, this system also generates surplus electricity. The estimated levelised cost of methanol production is 596.26 €/t (cost reported in $ in the reference; for comparison, the equivalent value in € was calculated using an average exchange rate of 1 $ = 0.85 €).

Akbary et al. [31] present a system for seasonal energy storage and e-methanol production from biomass. The system generates biogas for electricity production during periods of low renewable energy supply and captures CO_2_ for reuse. Hydrogen is produced via solid oxide electrolysis using surplus electricity. The estimated methanol cost is 900 €/t in 2023, potentially decreasing to 500 €/t by 2040. With CO_2_ taxes, this system may become competitive with fossil-based methanol. Galinova et al. [32] proposed a method for identifying optimal locations for e-methanol production by analysing the transport of CO_2_, H_2_, and electricity. Their findings indicate that transporting CO_2_ to regions with access to renewable electricity is the most cost-effective solution. Alternative options for transporting H_2_ or electricity are projected to be 3–13% more expensive by 2030 and 5–15% more expensive by 2050, when economic, logistical, and social factors are considered in site selection. Table 2 presents a summary of the literature review on methanol synthesis via CO_2_ hydrogenation.

### 1.5. New Uses of Methanol

Beyond these techno-economic assessments of new low-carbon or renewable methanol routes, other works examine emerging uses of methanol as an energy carrier and fuel. Methanol is attracting increasing attention because it is liquid under ambient conditions, can be stored and transported using comparatively mature infrastructure, and can serve either as a final fuel or as an intermediate hydrogen carrier. Zhao [33] and Liu [34] study methanol as a vector for hydrogen production, evaluating solar-assisted methanol steam reforming and proposing optimisation strategies. In the transport sector, methanol is also being considered as a promising alternative fuel for maritime applications, where fuel storage, bunkering infrastructure and compatibility with dual-fuel engines are key deployment factors. Körberg [35] compares several marine fuels across ship types and concludes that methanol is the lowest-cost option. These emerging applications strengthen the case for scalable renewable methanol production routes that are not only low-carbon but also compatible with existing fuel logistics and future demand growth.

### 1.6. Research Gap

Taken together, this literature shows a broad portfolio of fossil-, biomass-, and electricity-based routes to low-carbon methanol, as well as a wide range of potential CO_2_ sources. However, most studies focus on specific combinations of feedstock, CO_2_ source and plant configuration, often at the scale of individual projects, and typically treat biomethanol and e-methanol routes separately. In particular, CO_2_/H_2_ synthesis is mainly associated with e-methanol production using electrolytic hydrogen, whereas its integration into biomethanol routes using biohydrogen from biomethane reforming has received limited attention. This is especially relevant because syngas-based biomethanol production also requires an external biogenic CO_2_ supply.

Only a few works systematically compare alternative biomethanol pathways that use biomethane as the central feedstock, and even fewer analyse how such routes perform in terms of efficiency, CO_2_ self-sufficiency and cost under realistic national biomethane potential. In particular, the role of biomethane as a sole carbon and hydrogen source, and the extent to which methanol production can be decoupled from dedicated biogenic CO_2_ supply chains, remain insufficiently explored.

This study addresses this gap by proposing and evaluating four biomethanol production routes that combine conventional CO/H_2_ methanol synthesis with CO_2_/H_2_ synthesis. The novelty of this work lies not only in the use of biomethane as feedstock, which has been previously analysed, but in the design of CO_2_-self-sufficient routes in which biohydrogen is produced internally through steam biomethane reforming with carbon capture and utilisation. Unlike e-methanol pathways, the proposed CO_2_/H_2_ synthesis does not require electrolytic hydrogen. Unlike conventional biomethanol pathways, the proposed routes can avoid external biogenic CO_2_ supply chains by reusing the captured biogenic CO_2_ internally as methanol feedstock.

The first route, used as the baseline, mirrors the conventional methane-based process (syngas route) but replaces fossil methane and CO_2_ with renewable counterparts, requiring an external biogenic CO_2_ supply. The other three novel routes explicitly address the CO_2_ supply constraint and achieve CO_2_ self-sufficiency. This is extremely important because the CO_2_ required for renewable methanol should be biogenic, which may create logistical challenges associated with CO_2_ collection, purification, compression, and transport. By combining the two conventional methanol synthesis pathways (CO_2_/H_2_ and CO/H_2_) in different configurations, the proposed routes rely solely on biomethane as both a carbon and a hydrogen source, thereby overcoming constraints associated with biogenic CO_2_ availability and providing a structured framework to assess biomethane-based biomethanol at the scale of a national system.

Within this context, recent contributions by Rinaldi et al. [7] and Niu et al. [13] on biogas reforming are particularly relevant but remain limited to single residue streams and do not provide a comparative techno-economic assessment across multiple feedstocks and reforming configurations. To the best of the authors’ knowledge, this is the first study to directly compare the techno-economic performance of biomethanol production from several waste-derived biomethane streams, while also assessing alternative biomethane-based routes in terms of efficiency, CO_2_ self-sufficiency, and LCOM.

The present study fills this gap by assessing four biomethane-reforming pathways and their overall techno-economic performance. The analysis first evaluates the methanol production efficiency of each pathway to identify the most effective process configurations, explicitly accounting for feedstock availability. An economic assessment is then carried out to estimate the LCOM under different scenarios, focusing on the two most efficient routes and on various combinations of biomethane feedstocks. Key cost drivers are included, such as capital investment, feedstock supply costs, operating and maintenance expenses and other relevant economic factors. This provides a comprehensive evaluation of the economic feasibility and deployment potential of producing CO_2_ self-sufficient biomethanol using the proposed routes.

## 2. Methods

The classical methanol production from natural gas (syngas route) is based on the CO/H_2_ synthesis described by Equation (1) [1]. Hydrogen and carbon monoxide are obtained from natural gas (methane) via steam methane reforming (SMR), as shown in Equation (2), followed by a reverse water–gas shift (RWGS) reaction, Equation (3), to achieve the stoichiometry required by Equation (1). When natural gas is replaced by biomethane and biogenic CO_2_ is used, the product is biomethanol. This process is referred to as route 1 in this study and is taken as the baseline.

(1) $$CO+2H_{2}\to CH_{3}OH$$

(2) $$CH_{4}+H_{2}O\to CO+3H_{2}$$

(3) $$CO_{2}+H_{2}\to CO+H_{2}O$$

The proposed route 2 follows the CO_2_/H_2_ synthesis (CO_2_ hydrogenation) given in Equation (4), currently employed to produce e-methanol [1]. Hydrogen and carbon dioxide are obtained from SMR of biomethane (Equation (2)), followed by a water–gas shift reaction (WGS), Equation (5). Carbon capture and utilisation (CCU) is applied after the SMR process to separate CO_2_ and H_2_. All the hydrogen produced is consumed on site, so the main products are biomethanol and biogenic CO_2_. The separated CO_2_ can either be commercialised or stored, with the latter option leading to negative emissions.

(4) $$CO_{2}+3H_{2}\to CH_{3}OH+H_{2}O$$

(5) $$CO+H_{2}O\to CO_{2}+H_{2}$$

Routes 3 and 4 are combinations of the two previous ones. Since route 1 requires biogenic CO_2_, route 3 generates this CO_2_ from biomethane via SMR + WGS + CCU, with hydrogen obtained as a by-product. Route 4 combines route 1 and 2: the biogenic CO_2_ produced and captured in route 2 is used as the CO_2_ feed for route 1. In summary, only route 1 requires external CO_2_ as feedstock; in the other routes, CO_2_ is self-produced. As for by-products, route 2 yields biogenic CO_2_ whereas route 3 yields biohydrogen. Table 3 summarises the main characteristics of the four routes. As can be seen, route 1 corresponds to the conventional pathway currently used for methanol production from natural gas, with biomethane replacing natural gas in the present analysis. Route 2 is a variation of the current approach for producing green methanol as a renewable fuel of non-biological origin (RFNBO), based on hydrogen from renewable electrolysis (e-H_2_) and captured CO_2_. In the present study, e-H_2_ is replaced by biohydrogen produced through biomethane reforming, while the biogenic CO_2_ generated during the process is captured and utilised. Thus, both pathways are based on well-stablished technologies in which biomethane is introduced as a renewable feedstock. The main novelty of this work lies in routes 3 and 4, both of which incorporate route 1 and rely on CO_2_ internally supplied through biomethane reforming. In route 3, reforming is used solely for hydrogen production in combination with CCS, whereas route 4 integrates a complete methanol production facility based on route 2.

The CO_2_ captured at selected stages of the process is assumed to reach the purity required for its subsequent use in methanol synthesis. A similar approach is commonly applied in the production of other chemicals, such as urea [36].

Biomethane is produced by upgrading cleaned biogas. Biogas is a renewable gas generated through the anaerobic digestion of organic waste, including the organic fraction of municipal solid waste, wastewater treatment plant sludge, agri-food waste, agricultural waste, intermediate crops, and manure. Raw biogas consists mainly of methane and carbon dioxide, together with a range of impurities that are removed during the cleaning stage. The resulting cleaned biogas is therefore composed primarily of methane and carbon dioxide. Although cleaned biogas may be used directly for local combustion, it can also be upgraded by removing CO_2_ to produce biomethane of sufficient quality for injection into the natural gas grid. The separated CO_2_ is itself a valuable by-product, as its biogenic origin allows it to be used as a feedstock for the production of renewable fuels. Detailed descriptions of biogas cleaning and upgrading technologies, together with their associated costs, can be found in [37]. As reported in previous studies [7,11,15], biogas, and more specifically biomethane, can be used as a substitute for natural gas in methanol production, thereby enabling the transition from fossil-based methanol to biomethanol.

The LCOM has been assessed, including investment, operation, maintenance and feedstock costs, according to Equations (6)–(9), where the subscript “*x*” stands for either CH_4_ or CO_2_. The capital recovery factor (CRF) is given by Equation (10). The weighted average capital cost (wacc) has been set to 7.5%, the project lifetime (N) to 25 years, and the operation and maintenance factor (fom) to 5% [1].

(6) $$LCOM=CAPEX+{OPEX}_{om}+{OPEX}_{{CH}_{4}}+{OPEX}_{{CO}_{2}}$$

(7) $$CAPEX=\left(\frac{INV}{M}\right)\cdot CRF$$

(8) $${OPEX}_{om}=\left(\frac{INV}{M}\right)\cdot f_{om}$$

(9) $${OPEX}_{x}=T_{x}\cdot C_{x}$$

(10) $$CRF=\frac{wacc\cdot {\left(1+wacc\right)}^{N}}{{\left(1+wacc\right)}^{N}-1}$$

The nominal plant capacity is set to 200,000 t MeOH per year [1]. Investment costs for route 1 (Equation (11)) and for CO_2_/H_2_ methanol synthesis (Equation (12)) have been derived from [1], whereas the costs for SMR + WGS + CCU (Equation (13)) have been determined based on Yagüe et al. [38].

(11) $${INV}_{R1}=19,274\cdot M^{0.7418}$$

(12) $${INV}_{{CO}_{2}/H_{2}}=404,524\cdot M^{0.5198}$$

(13) $${INV}_{SMR+WGS+CCU}=56,884\cdot H^{0.713}+8347\cdot H^{0.8592}$$

Costs for biogas production, upgrading and injection into the natural gas grid have been taken from Flotats and Feliu [37]. The biomethane potential is based on data from [39]. The cost of biogenic CO_2_ ($T_{{CO}_{2}}$) is assumed to be 125 €/t, sourced from the biogas upgrading process [38]. The CO_2_ is transported by pipeline from the biomethane upgrading facilities. Transport costs are based on the FECM/NETL CO_2_ Transport Cost Model (CO2_T_COM), a tool developed by the U.S. Department of Energy’s Office of Fossil Energy and Carbon Management (FECM) [40]. For the present study, the specific transport cost is extrapolated from [41], where the model was applied to the Spanish context, obtaining a value of 71 €/t CO_2_ for a total mass flow rate of 68 kt CO_2_/year, corresponding to the requirements of a methanol plant with an annual production capacity of 200 kt/year.

Regarding feedstock, four combinations of substrates have been considered for biomethane production, as shown in Table 4, where OFMSW stands for the organic fraction of municipal solid waste and WWTP refers to wastewater treatment plant. Combination A represents the projected composition of the future biomethane mix in the grid, based on Sedigas’ forecasts [39]. This combination results in a cost similar to expected market values. The remaining combinations aim to reduce biomethane costs by using the cheapest substrates—landfill gas and OFMSW. Specifically, combination B includes both substrates, whereas combinations C and D consider each one individually. Biomethane potential and feedstock costs for all combinations have been also included in Table 4.

The availability of biogenic CO_2_ for route 1 has been assessed based on the CO_2_ released during the biomethane upgrading process. Assuming a CO_2_ composition of 35% to 45% in clean biogas, it is estimated that 1 GWh-HHV of biogas produces between 95.81 and 145.57 t CO_2_. Although this amount is sufficient to meet the CO_2_ demand for methanol production, as shown in Table 5, its supply poses a significant logistical challenge because biomethane production is geographically dispersed. Consequently, the development of a CO_2_ transport network would be required, and sufficiently large flow rates would be needed to keep transport costs within reasonable limits. In the present study, a transport cost of 71 €/t CO_2_ is adopted, corresponding to the CO_2_ demand of a 200 kt/year methanol plant, i.e., approximately 68 kt CO_2_/year. However, [41] reports transport costs exceeding 745 €/t CO_2_ for flow rates below 9.4 kt CO_2_/year. The present work addresses this limitation by internally producing the required CO_2_, thereby avoiding the need for the construction and operation of a dedicated CO_2_ transport network. To quantify the degree of CO_2_ self-sufficiency of each route, a CO_2_ self-sufficiency ratio, $SSR_{{CO}_{2}}$, has been defined (Equation (14)) as the ratio between the internally available biogenic CO_2_ stream suitable for methanol synthesis and the CO_2_ required by the methanol synthesis step. It should be noted that the internally available CO_2_ includes only the fraction captured at the assumed capture efficiency (90%) and at process locations where it can be suitably recovered [42]. A value of $SSR_{{CO}_{2}}=0$ indicates full dependence on external CO_2_ supply, $SSR_{{CO}_{2}}=1$ indicates exact CO_2_ self-sufficiency, and $SSR_{{CO}_{2}}>1$ indicates a surplus of internally generated biogenic CO_2_. This ratio is intended solely to assess the extent to which the CO_2_ demand of the methanol synthesis process can be met internally and should not be interpreted as a carbon balance for the overall process.

(14) $$SSR_{{CO}_{2}}=\frac{CO_{2,internal}}{CO_{2,required}}$$

## 3. Results

### 3.1. Mass Balance

Route 1 uses SMR (Equation (2)) and an RWGS reaction (Equation (3)) to obtain the H_2_/CO ratio of 2:1 required for the methanol synthesis reaction. Taking the reforming reaction as the basis, the RGWS reaction is adjusted accordingly. In order to maintain the thermal conditions in the reformer, an additional amount of methane must be burned. Assuming an SMR efficiency of 75.9% on a lower heating value basis [42], the combustion equation is adjusted. Finally, the resulting H_2_ and CO feed the CO/H_2_ methanol synthesis, for which an efficiency of 100% has been assumed, according to Kuczynski et al. [43] and Stahl et al. [44].

In route 2, SMR with CCU provides the H_2_ and CO_2_ required by the CO_2_/H_2_ methanol synthesis. Again, the reforming reaction is taken as the basis, followed by the WGS reaction. Additional methane is combusted to maintain the required thermal conditions, assuming an efficiency of 69.1%, based on the lower heating value, due to the carbon capture process [39]. Assuming a CCU efficiency of 90% [39], the captured CO_2_ is split into three streams, as the hydrogen is taken as the limiting reactant. Part of this captured CO_2_ and all of the H_2_ are sent to the CO_2_/H_2_ synthesis, for which an efficiency of 100% has been assumed, in accordance with Vaquerizo et al. [45]. Overall, 187.51 kg of H_2_ from the SMR + WGS + CCU plant are required to produce 1 t of MeOH.

**Figure 1:**
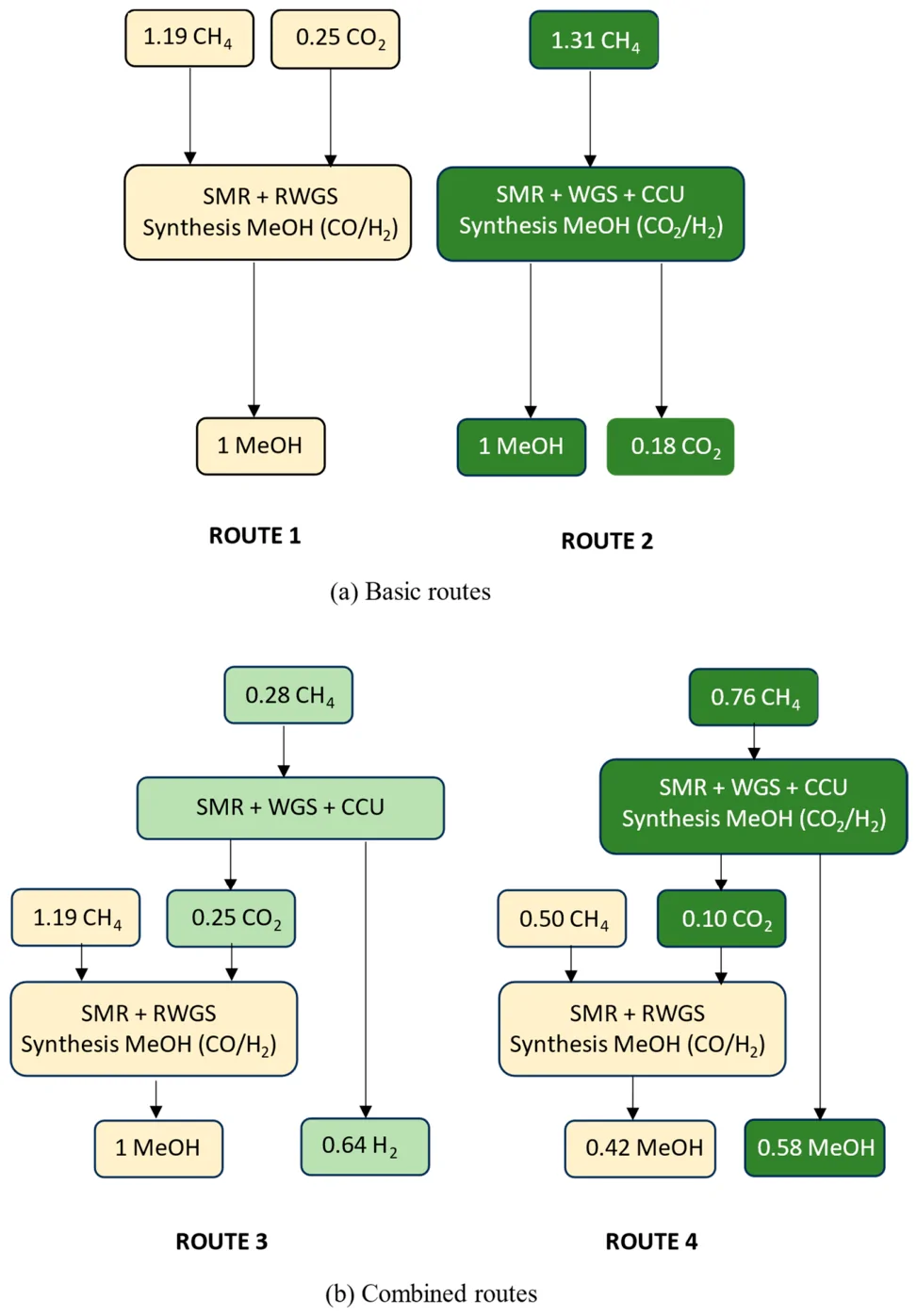
Mass balance (in molar basis) of the routes proposed.

**Figure A1:**
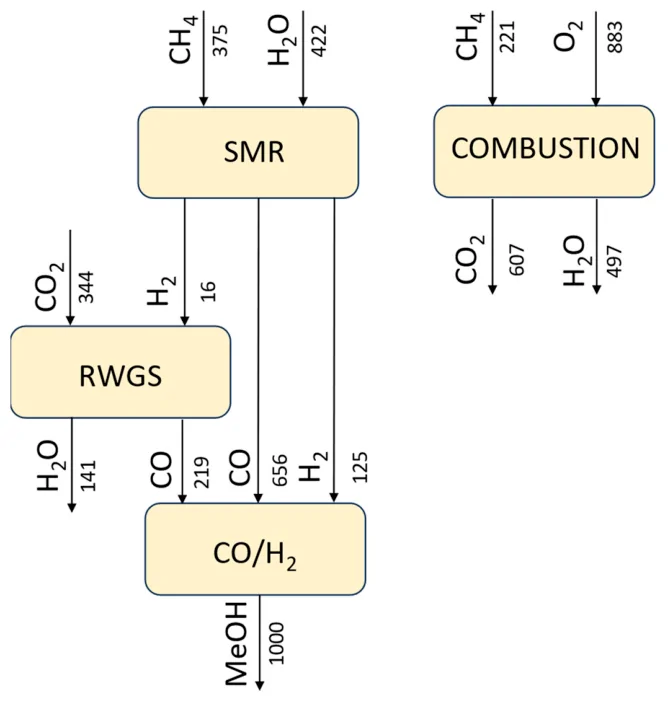
Mass balance (kg) of route 1.

**Figure A2:**
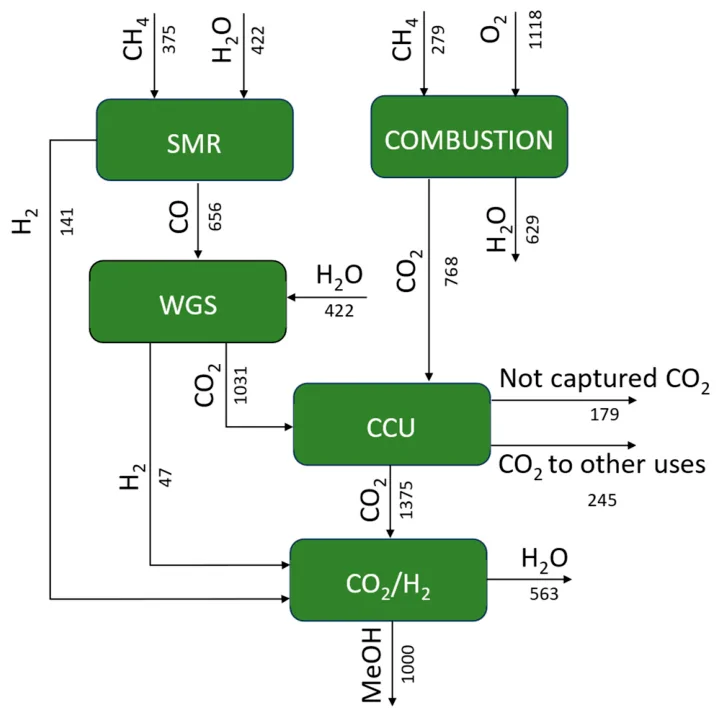
Mass balance (kg) of route 2.

**Figure A3:**
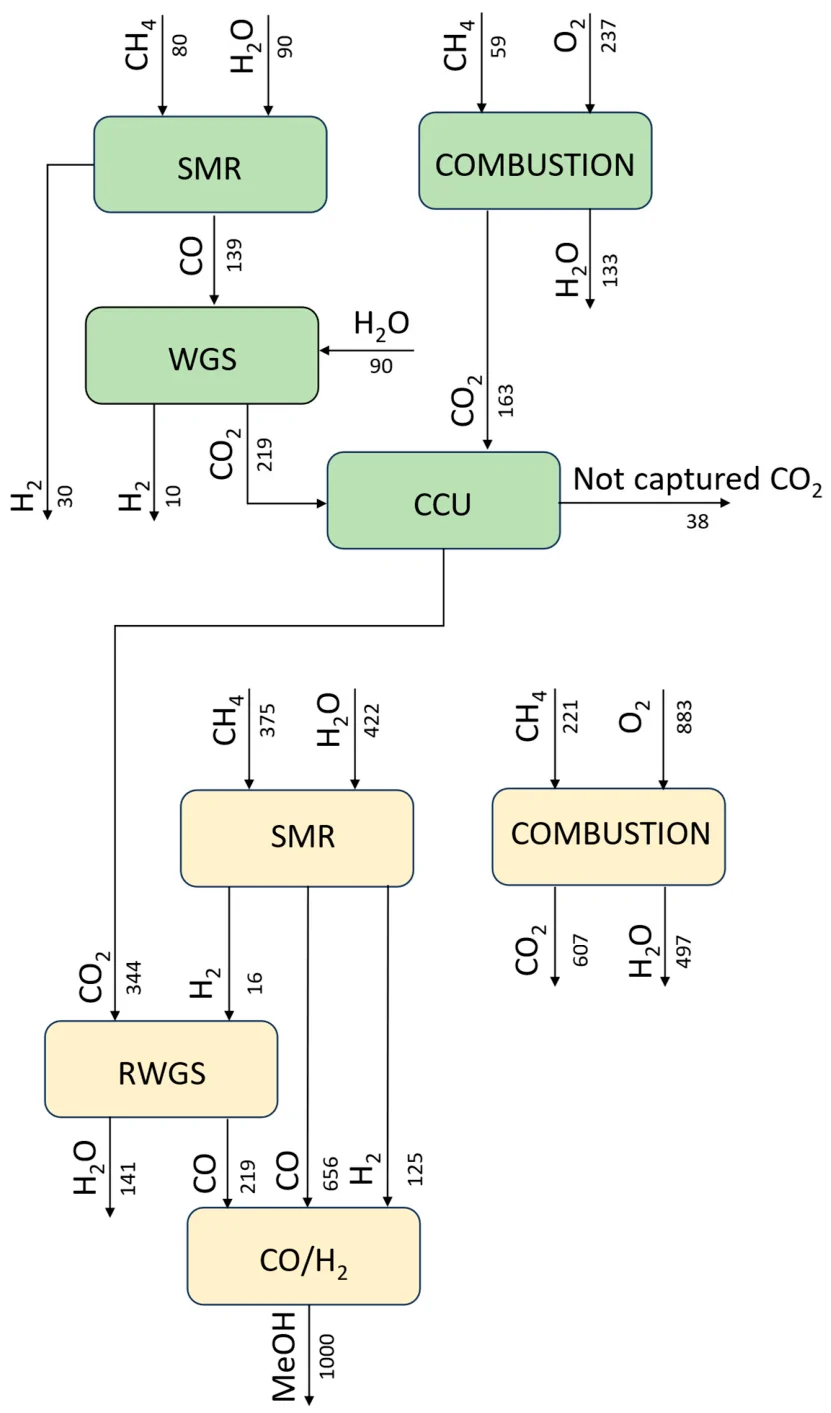
Mass balance (kg) of route 3.

**Figure A4:**
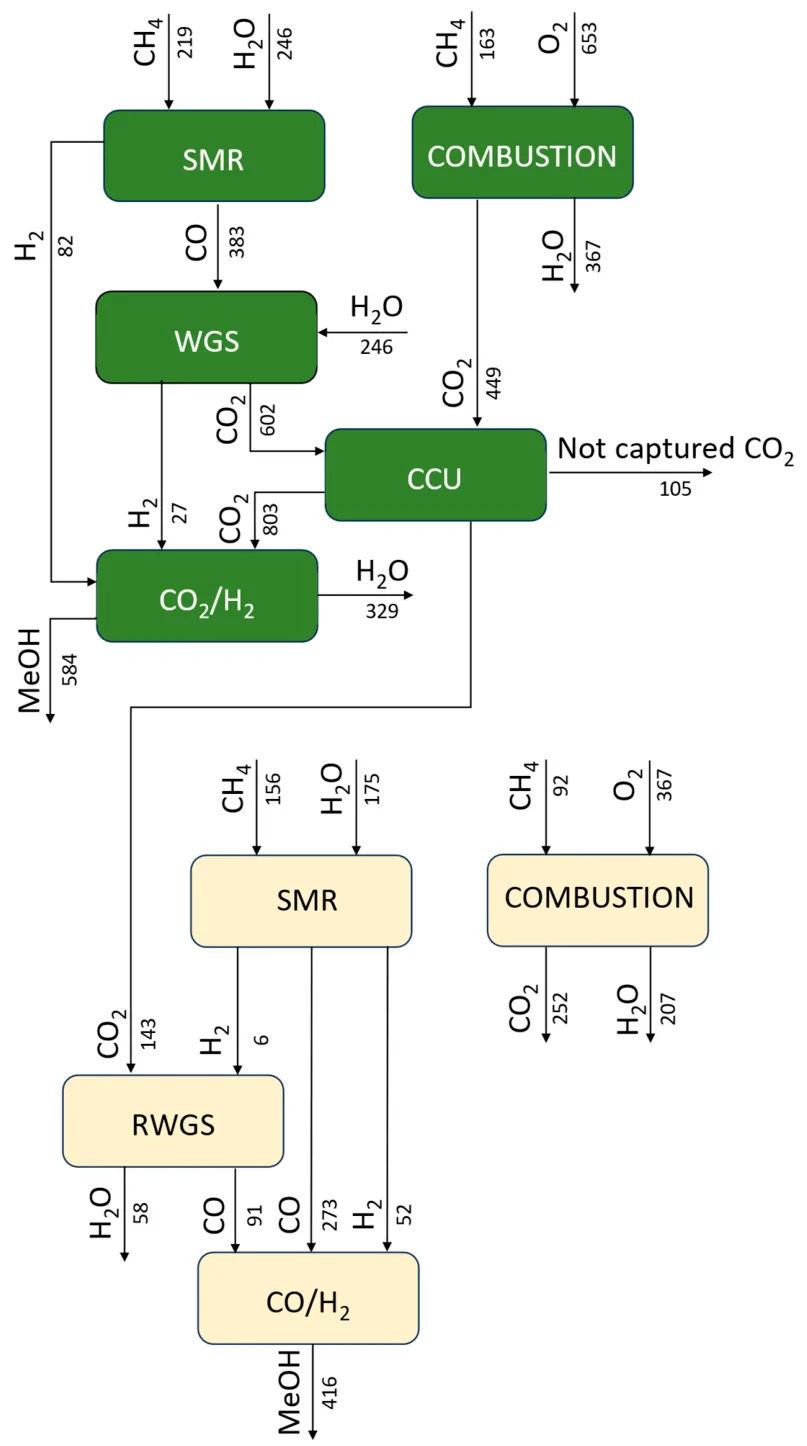
Mass balance (kg) of route 4.

In route 3, the SMR + WGS + CCU process is used to supply the CO_2_ needed by route 1. The SMR reaction from route 1 is taken as the basis, and the SMR + WGS + CCU reactions are adjusted to provide the CO_2_ required in route 1 (1/3 CO_2_ per 4/3 MeOH). In this case, only 39.8 kg H_2_ are produced as a by-product per tonne of methanol. Route 4 combines routes 2 and 1 by using the CO_2_ surplus from route 2 to feed route 1. Figure 1 shows the interconnection between routes and the molar basis mass balance, using 1 mol of MeOH as the reference. Table A1 and Figure A1, Figure A2, Figure A3 and Figure A4 in Appendix A summarises the processes and reactions involved in each route.

According to Figure 1, the balance between feedstock and products for the different routes is summarised in Table 5, along with the evaluation of the CO_2_ self-sufficiency ratio. Route 1 is identified as the most efficient option to produce methanol, with a biomethane consumption of 9.21 MWh-HHV/t MeOH (108.6 t MeOH/GWh-HHV of biomethane), but it requires 344 kg CO_2_ per tonne of methanol produced. Considering that at least 95.81 t CO_2_ are removed from biogas to obtain 1 GWh-HHV of biomethane [38], the CO_2_ available from the upgrading process to produce 1 tonne of methanol via route 1 is at least 882 kg CO_2_, that is, more than twice the required amount. Thus, the limiting factor for biomethanol production through route 1 is the availability of biomethane, rather than biogenic CO_2_. In practice, however, the lack of infrastructure to transport CO_2_ from biomethane production units to methanol plants is a challenge, which is addressed by the other three routes. As shown in Table 5, the most efficient of the three CO_2_ self-sufficient options is route 4, with a biomethane consumption of 9.74 MWh-HHV/t MeOH (102.7 t MeOH/GWh-HHV of biomethane). Consequently, routes 1 and 4 have been chosen for the cost and production potential comparison in Spain.

### 3.2. Levelised Cost of Methanol

**Figure 2:**
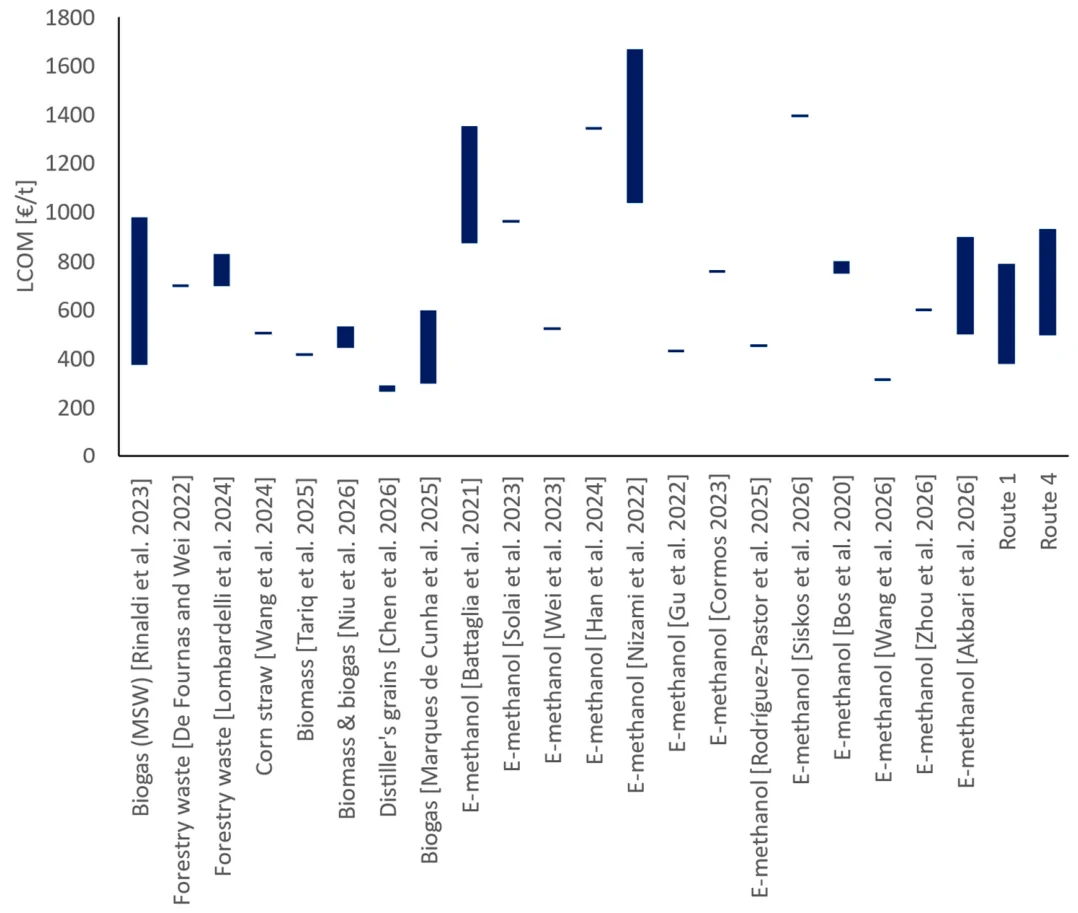
Comparison of methanol production costs for routes 1 and 4 with literature data [7,9,10,11,12,13,14,15,17,18,19,20,21,22,23,24,25,27,28,30,31].

Table 6 presents the breakdown of LCOM for routes 1 and 4, including investment, maintenance and feedstock costs as a function of the substrate mix used to produce biomethane. It is observed that the costs for route 4 (self-produced CO_2_) are 14 to 23% higher than those for route 1 (baseline). In any case, methanol costs are strongly influenced by feedstock, which accounts for 47% to 72% of total LCOM; in route 1, the cost of biogenic CO_2_ contributes a further 8% to 17%. These values are consistent with those reported in [1], which reports biomethanol costs of 400–800 $/t for 2020–2025, decreasing to 300–650 $/t in 2035–2040 for biomethanol from MSW-derived biogas (substrates B and C). Figure 2 compares the LCOM results from this study with literature data. The reviewed studies span different technologies, feedstocks, plant scales, and LCOM calculation methods, which explains the wide spread of reported costs. The articles included in Figure 2 cover several production routes: biogas reforming [7,13,15], biomass gasification [9,10,11,12,13,14], and e-methanol production in the remaining works. The LCOM values obtained here for both biomethane reforming routes are consistent with those reported by Rinaldi et al. [7], where biomethanol is also produced from biogas. Literature LCOM values for biomethanol from biomass gasification fall within the range of routes 1 and 4. In contrast, e-methanol shows larger cost dispersion and generally higher LCOM, mainly due to the high cost of renewable hydrogen.

Table 6 also summarises the biomethanol potential and the LCOM of routes 1 and 4 as a function of substrate composition, determining the number of 200 kt/year methanol plants that could be installed in Spain if the entire biomethane potential were exploited. Substrate composition A exhibits, by far, the highest potential (supporting more than 70 plants in route 1 and 69 in route 4), but it also has the highest LCOM due to the logistical challenge of using diverse substrates. Conversely, the lowest-cost substrate (100% landfill gas) offers the smallest potential, supporting only 4 plants. A good compromise between potential and cost is achieved with composition B (biomethane from municipal solid waste and landfill gas), which in route 4 yields about 1.6 Mt/year of biomethanol at a cost of 608 €/t. A substantial difference can be observed between the potential number of plants associated with substrate A and those associated with the other substrates. Since the methanol production capacity is fixed at 200 kt/year for all plants, this difference is mainly attributable to the considerably greater potential of substrate A (more than 135 TWh) compared with that of the other substrates (8 to 17 TWh).

In Table 6, the CAPEX of route 4 is broken down into the contributions associated with routes 1 and 2. The contribution corresponding to route 1 is similar to that of route 1 operated independently, when normalised by the amount of methanol produced. In contrast, the route 2 contribution is considerably more CAPEX-intensive. The resulting additional cost, estimated at 148 €/t of methanol when both CAPEX and O&M costs are considered, is not offset by the 67 €/t saving associated with CO_2_ self-sufficiency.

The main limitation of this study is the current state of development of the biomethane sector, since route 4 requires 1.95 TWh of biomethane per 200 kt/year methanol plant. At present, biomethane production in Spain is about 528 GWh/year [46], whereas the technical potential is estimated at 163 TWh/year [39]. Thus, the biomethanol production levels projected in this work are therefore only attainable in the medium term, once biomethane plants have been widely deployed. In addition, route 4 requires carbon capture in the SMR.

**Figure 3:**
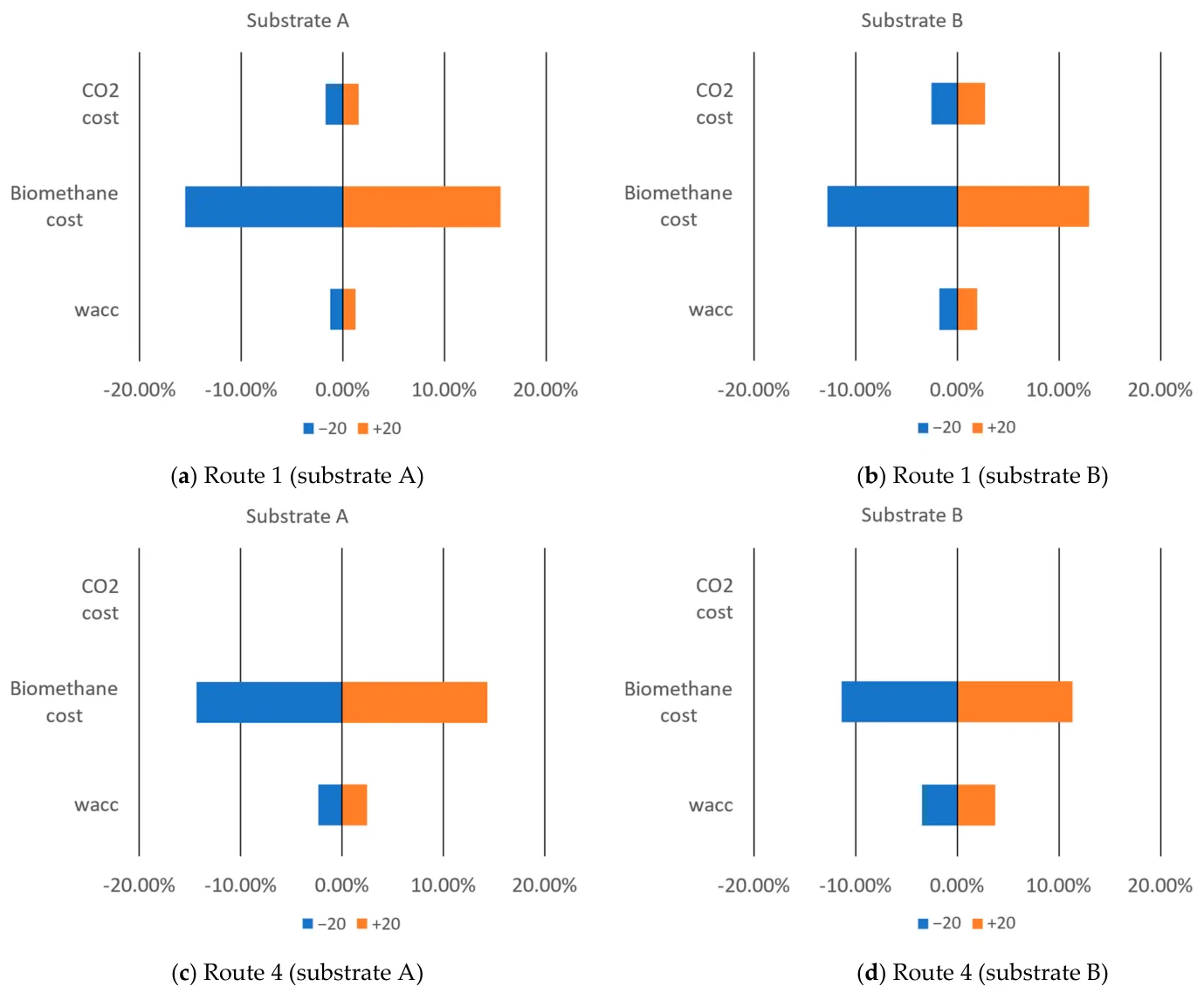
Sensitivity analysis of the LCOM to feedstock costs (biomethane and CO_2_) and CAPEX (wacc) for substrates A and B in routes 1 and 4.

**Figure 4:**
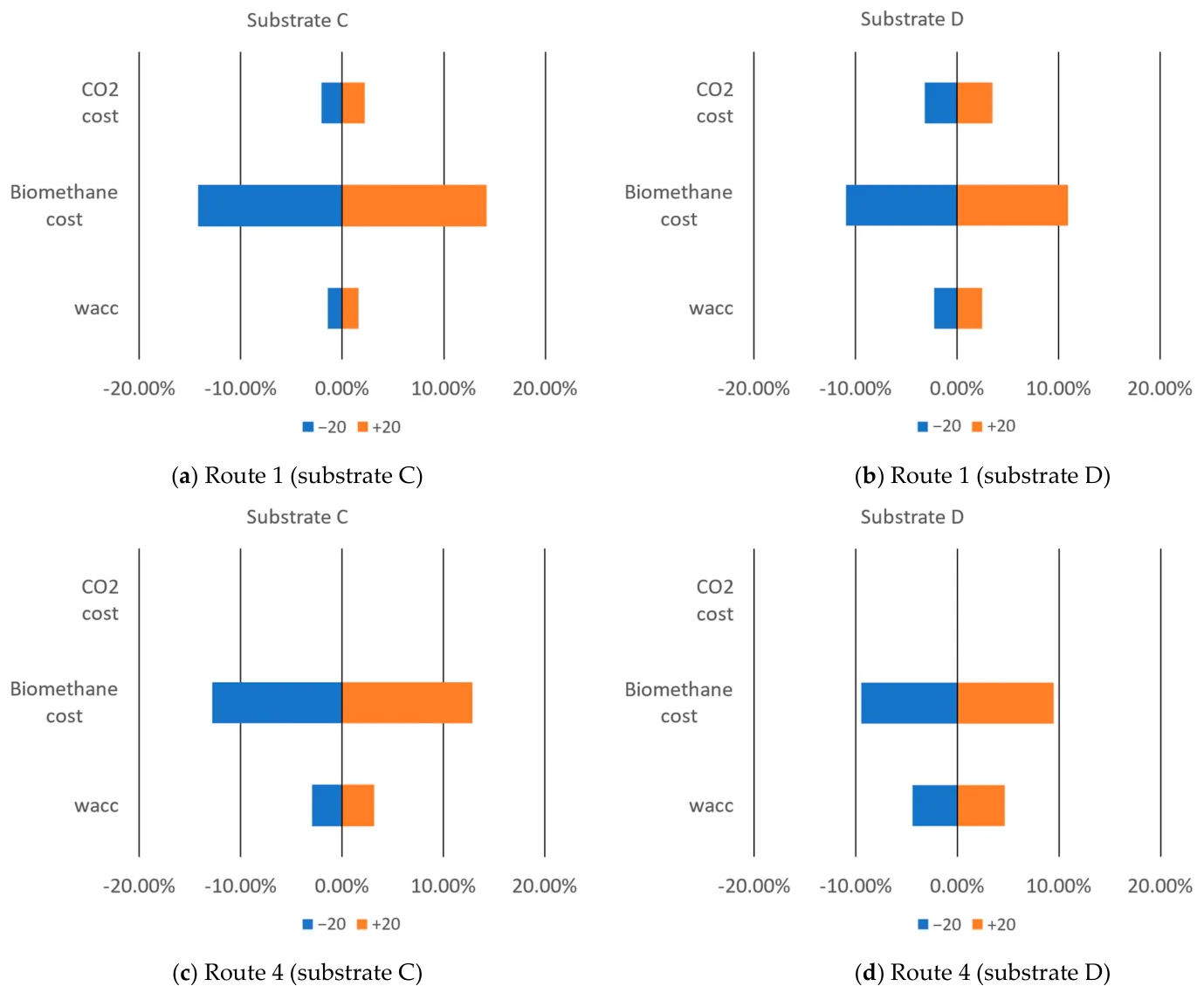
Sensitivity analysis of the LCOM to feedstock costs (biomethane and CO_2_) and CAPEX (wacc) for substrates C and D in routes 1 and 4.

Table 6 shows that biomethane cost is the largest contributor to the LCOM. However, route 4 exhibits a substantially higher CAPEX than route 1, making its LCOM more sensitive to the wacc. In route 1, CO_2_-related costs, including both acquisition and transport, also make a significant contribution. Figure 3 and Figure 4 present a sensitivity analysis of these parameters. The results show that, in route 1, the LCOM is more sensitive to CO_2_ costs than to wacc. By contrast, in route 4, the greater influence of wacc reduces the relative contribution of biomethane cost to the overall sensitivity, although biomethane remains the main cost driver in both routes.

## 4. Discussion

Methanol is a key building block for chemical products and a promising alternative fuel for shipping. However, its conventional production from fossil fuels is a major source of CO_2_ emissions. This study has examined four routes to produce biomethanol from biomethane in Spain, with the aim of identifying process configurations that are both efficient and compatible with realistic biomethane potentials. Although the classical route (route 1) achieves the highest conversion efficiency, with 108.6 t MeOH per GWh-HHV of biomethane, it relies on an external supply of biogenic CO_2_ and is therefore constrained by the availability and logistics of separate CO_2_ streams.

To address this limitation, three alternative routes were proposed, all of which internally produce biogenic CO_2_ through biomethane reforming and carbon capture. Among these, route 4 offers the best compromise between efficiency and process integration, achieving 102.7 t MeOH per GWh-HHV of biomethane while remaining fully CO_2_ self-sufficient. For biomethane derived from a mixture of municipal solid waste and landfill gas, route 4 yields an LCOM of 608 €/t and could support up to eight 200 kt/year plants in Spain. This configuration relies on mature technologies—steam methane reforming, conventional methanol synthesis and post-combustion carbon capture—and reuses the captured CO_2_ within the process. Although this integration increases the LCOM by 14–23% compared with route 1, the premium can be interpreted as the price of CO_2_ feedstock self-sufficiency: route 4 internally produces and reuses the biogenic CO_2_ required for methanol synthesis, thereby eliminating the need for external CO_2_ supply from geographically dispersed biogas-upgrading plants and avoiding dedicated CO_2_ collection, purification, compression, and transport infrastructure.

The economic analysis confirms that feedstock cost is the dominant driver of LCOM for both the baseline and self-sufficient routes. The sensitivity analysis shows a greater influence of the wacc in route 4, owing to the higher share of CAPEX in this route compared with route 1. Nevertheless, feedstock cost remains the main driver, especially in route 1, where both biomethane and CO_2_ contribute significantly to the LCOM. While route 1 remains the most efficient in energetic terms, route 4 demonstrates that a biomethane-only concept, in which biomethane is the sole carbon and hydrogen source, can deliver biomethanol at costs that are competitive with other renewable methanol options reported in the literature, especially when low-cost waste-derived biomethane is available. In this sense, the proposed framework shows that waste streams such as MSW-derived biogas and landfill gas can underpin a scalable biomethanol supply, contributing simultaneously to waste management, renewable fuel production, and industrial decarbonisation.

At the system level, the results suggest that Spain could, in the medium term, develop a portfolio of biomethanol plants based on the most favourable combinations of waste-derived biomethane, with route 4 as a robust reference configuration. Such plants could supply a non-negligible fraction of future methanol demand for the chemical and maritime sectors while remaining anchored in domestic resources. However, the main limitation of the present study is biomethane availability: scaling biomethanol production to the levels identified here will require a substantial expansion of biomethane production, upgrading, and injection infrastructure, together with policy instruments that prioritise waste-based feedstocks. Overall, this study provides a structured techno-economic basis for designing and scaling biomethane-based biomethanol production in Spain and offers a transferable methodology that can be applied to other countries with significant untapped biomethane potentials.

A natural next step in this research would be to include a comprehensive life cycle analysis (LCA) to evaluate not only the carbon footprint, but also other environmental impacts. Similar analyses have been carried out by other authors, such as Tariq et al. [12] and Gholizadeh et al. [47]. The former analysed biomethanol production via biomass gasification, whereas the latter investigated an integrated system combining biomethanol and biomethane production with high-temperature electrolysis, showing that the overall environmental impact strongly depends on the renewable electricity source considered. However, these results cannot be directly extrapolated to the routes proposed in the present study. A tailored LCA would therefore be required to assess their overall environmental performance, although such an analysis lies beyond the scope of the present work.

## Figures and Tables

**Table 1:** Summary of reviewed studies on methanol synthesis via the syngas route.

Author	Feedstock	Route	LCOM
Ren et al. [4]	Natural gas and CO_2_ from a power plant	SMR	100 $/t
Lim et al. [5]	Natural gas and electrolytic H_2_	Tri-reforming	488.01–849.21 $/t
Arnaiz del Pozo et al. [6]	Natural gas	SMR-ATR; ATR-HTER or GSR	252–268.4 €/t
Rinaldi et al. [7]	Biogas from OFMSW	SMR	378–981 €/t
Kgwedi et al. [8]	Glycerol (Diesel subproduct)	SMR	850–981 $/t
De Fournas and Wei [9]	(a) Forestry residues; (b) forestry residues and electrolytic H_2_	(a) Gasification; (b) gasification and H_2_ injection	(a) 940–1004 $/t (b) 695–701 $/t
Lombardelli et al. [10]	Forestry biomass	Gasification; gasification and H_2_ addition	700–831 €/t
Wang et al. [11]	Corn straw	Gasification	502.0 $/t
Tariq et al. [12]	Biomass; renewable electricity and CO_2_ captured from syngas	Gasification and CO_2_ hydrogenation	413 €/t
Niu et al. [13]	(a) Natural gas; (b) biomass; (c) biogas from agricultural residues	(a) SMR; (b) gasification; (c) SMR	445.2–533.23 $/t
Chen et al. [14]	Distiller’s grains	(a) Gasification; (b) SMR of AD and gasification of digestate	(a) 264.73 $/t (b) 292.59 $/t
Marques da Cunha et al. [15]	Biogas	Bi- and tri-reforming	300–600 $/t

**Table 2:** Summary of reviewed studies on methanol synthesis via CO_2_ hydrogenation.

Author	Feedstock	Route	LCOM
Arnaiz del Pozo et al. [6]	PV or wind electricity and CO_2_ from DAC or pipeline	CCU (CO_2_) and H_2_ Electrolytic	471.6–784.9 €/t in 2050
Battaglia et al. [17]	Hydropower electricity and CO_2_ from coal-fired power plant	CCU (CO_2_) and H_2_ Electrolytic	874–1356 €/t
Sollai et al. [18]	Renewable electricity surpluses and CO_2_ from fossil fuel thermal plant	CCU (CO_2_) and H_2_ Electrolytic	960 €/t
Wei et al. [19]	Renewable electricity and CO_2_ from power plant	CCU (CO_2_) and H_2_ Electrolytic	520 $/t
Ham et al. [20]	Wind and PV electricity and CO_2_ from oxy-fuel combined cycle	CCU (CO_2_) and H_2_ Electrolytic	1340 €/t
Nizami et al. [21]	PV-Grid and PV-battery electricity and CO_2_ from natural gas field processing	CCU (CO_2_) and H_2_ Electrolytic	PV-grid: 1040 $/t PV-battery: 1670 $/t
Gu et al. [22]	PV and wind electricity and CO_2_ from coal-based methanol production	CCU (CO_2_) and H_2_ Electrolytic	427 €/t
Cormos et al. [23]	PV and wind electricity and CO_2_ from industrial processes	CCU (CO_2_) and H_2_ Electrolytic	755 €/t
Rodriguez Pastor et al. [24]	PV electricity and CO_2_ from industries	CCU (CO_2_) and H_2_ Electrolytic	450 €/t
Siskos et al. [25]	PV electricity and CO_2_ from cement industry	CCU (CO_2_) and H_2_ Electrolytic	1390 €/t
Gelten et al. [26]	PV electricity and CO_2_ from biogas upgrading	CCU (CO_2_) and H_2_ Electrolytic	4059–4510 €/t
Bos et al. [27]	Wind electricity and CO_2_ from DAC	CCU (CO_2_) and H_2_ Electrolytic	750–800 €/t
Wang et al. [28]	Off-grid wind and PV electricity and CO_2_	CCU (CO_2_) and H_2_ Electrolytic	
Zhou et al. [30]	Renewable and waste heat recovery from MSW electricity; CO_2_ from CCU of MSW incineration	CCU (CO_2_) and H_2_ Electrolytic	
Akbary et al. [31]	Electricity from biogas and renewable grid surplus; CO_2_ from CCU of biogas power plant	CCU (CO_2_) and H_2_ Electrolytic	900 €/t in 2023 500 €/t expected in 2050

**Table 3:** Main characteristics of the four routes.

Route	Main Process	External CO_2_	By-Product	Role in Study
1	SMR + RWGS + CO/H_2_ synthesis	Yes	None	Baseline
2	SMR + WGS + CCU + CO_2_/H_2_ synthesis	No	CO_2_ surplus	CO_2_-producing route
3	SMR + CCU supplies CO_2_ to route 1	No	H_2_ surplus	Combined route
4	Route 2 CO_2_ surplus feeds route 1	No	None	Best self-sufficient route

**Table 4:** Combinations of substrates considered in the biomethane production [38,39].

Substrates	A	B	C	D
Landfill gas [%]	7	53		100
OFMSW [%]	6	47	100	
WWTP and Agri-Food Industry [%]	7			
Agriculture waste [%]	18			
Intermediate crops [%]	43			
Manure [%]	19			
Potential [TWh/year]	135.19	16.73	7.92	8.81
Feedstock cost [€/MWh-HHV]	69.33	35.95	48.77	24.43
$T_{{CH}_{4}}$ [€/t]	1071.6	555.64	753.79	377.59

**Table 5:** Balance of feedstocks and products for each route.

	Feedstocks			Products			$SSR_{CO2}$ [-]
Route	Biomethane [MWh-HHV]	$C_{{CH}_{4}}$ $\left[t_{{CH}_{4}}/t_{MeOH}\right]$	$C_{{CO}_{2}}$ $\left[t_{{CO}_{2}}/t_{MeOH}\right]$	MeOH [t]	H_2_ [kg]	CO_2_ [kg]	
1	9.21	0.5958	0.34	1	---	---	0
2	10.11	0.6544	---	1	---	245	1.178
3	11.35	0.7347	---	1	39.8	---	1
4	9.74	0.6300	---	1	---	---	1

**Table 6:** Comparison between routes 1 and 4 in Spain.

	Substrates	A	B	C	D
Route 1	CAPEX [€/t]	74	74	74	74
	OPEX OM [€/t]	41	41	41	41
	OPEX biomethane [€/t]	634	327	445	221
	OPEX CO_2_ [€/t]	67	67	67	67
	LCOM [€/t]	817	509	627	403
	Number of methanol plants (200 kt/year)	73	9	4	4
Route 4	CAPEX route 1 [€/t]	38.54	38.54	38.54	38.54
	CAPEX route 2 [€/t]	130.05	130.05	130.05	130.05
	OPEX OM [€/t]	94	94	94	94
	OPEX biomethane [€/t]	671	346	470	233
	OPEX CO_2_ [€/t]	0	0	0	0
	LCOM [€/t]	933	608	733	496
	Number of methanol plants (200 kt/year)	69	8	4	4

## Data Availability

The original contributions presented in this study are included in the article. Further inquiries can be directed to the corresponding author(s).

## Abbreviations

The following abbreviations are used in this manuscript:

Abbreviations		
AD	Anaerobic digestion	
ATR	Autothermal reforming	
CCU	Carbon capture and utilisation	
DAC	Direct air capture	
e-H_2_	Hydrogen from electrolysis	
FECM	Fossil Energy and Carbon Management	
GSR	Gas switching reformer	
HHV	Higher heating value	
HTER	Heat transfer exchanger reformer	
LCA	Life cycle analysis	
LCOM	Levelised cost of methanol	
MeOH	Methanol	
MSW	Municipal solid waste	
MTBE	Methyl tert-butyl ether	
OFMSW	Organic fraction of municipal solid waste	
OM	Operation and maintenance	
PV	Photovoltaic energy	
RFNBO	Renewable fuel of non-biological origin	
RWGS	Reversed water–gas shift	
SMR	Steam methane reforming	
WGS	Water–gas shift	
WWTP	Wastewater treatment plant	
Symbols		
Symbol	Concept	Unit
*CAPEX*	Capital expenditure of methanol	€/t
*CRF*	Capital recovery factor	1/year
$C_{x}$	Specific consumption of x-th feedstock	t x/t MeOH
$f_{om}$	Maintenance factor	p.u.
*H*	Annual hydrogen production	t
${INV}_{R1}$	Investment required by route 1	€
${INV}_{R2}$	Investment required by route 2	€
*LCOM*	Levelised cost of methanol	€/t
*M*	Annual methanol production	t
*N*	Project lifetime	years
${OPEX}_{x}$	x-th operational expenditure of methanol	€/t
${OPEX}_{om}$	Operational expenditure of maintenance	€/t
${SSR}_{{CO}_{2}}$	CO_2_ self-sufficiency ratio	p.u.
$T_{x}$	Tariff of x-th feedstock	€/t
*wacc*	Weighted average capital cost	p.u.

## Appendix A

**Table A1:** Reactions involved in the proposed routes.

	Process	Reaction
Route 1	SMR	$CH_{4}+H_{2}O\to CO+3H_{2}$
	Combustion	$0.5887\;CH_{4}+1.1774\;O_{2}\to 0.5887\;{CO}_{2}+1.1774\;H_{2}O$
	RWGS	$0.333\;CO_{2}+0.333\;H_{2}\to 0.333\;CO+0.333\;H_{2}O$
	CO/H_2_ synthesis	$1.3333\;CO+2.6666\;H_{2}\to 1.3333\;CH_{3}OH$
Route 2	SMR	$CH_{4}+H_{2}O\to CO+3\;H_{2}$
	Combustion	$0.745\;CH_{4}+1.49\;O_{2}\to 0.745\;{CO}_{2}+1.49\;H_{2}O$
	WGS	$CO+H_{2}O\to CO_{2}+H_{2}$
	CCU	0.1745 CO_2_ not captured CO_2_ 1.3333 CO_2_ to the methanol synthesis 0.2372 CO_2_ other uses or storage
	CO_2_/H_2_ synthesis	$1.3333\;CO_{2}+4\;H_{2}\to 1.3333\;CH_{3}OH+1.3333\;H_{2}O$
Route 3	SMR	$0.2122\;CH_{4}+0.2122\;H_{2}O\to 0.2122\;CO+0.6366\;H_{2}$
	Combustion	$0.1581\;CH_{4}+0.3162\;O_{2}\to 0.1581\;{CO}_{2}+0.3162\;H_{2}O$
	WGS	$0.2122\;CO+0.2122\;H_{2}O\to 0.2122\;CO_{2}+0.2122\;H_{2}$
	CCU	0.037 CO_2_ not captured CO_2_ 0.3333 CO_2_ to the RWGS
	SMR	$CH_{4}+H_{2}O\to CO+3H_{2}$
	Combustion	$0.5887\;CH_{4}+1.1774\;O_{2}\to 0.5887\;{CO}_{2}+1.1774\;H_{2}O$
	RWGS	$0.333\;CO_{2}+0.333\;H_{2}\to 0.333\;CO+0.333\;H_{2}O$
	CO/H_2_ synthesis	$1.3333\;CO+2.6666\;H_{2}\to 1.3333\;CH_{3}OH$
Route 4	SMR	$1.4051\;CH_{4}+1.4051\;H_{2}O\to 1.4051\;CO+4.2154\;H_{2}$
	Combustion	$1.0468\;CH_{4}+2.0937\;O_{2}\to 1.0468\;{CO}_{2}+2.0937\;H_{2}O$
	WGS	$1.4051\;CO+1.4051\;H_{2}O\to 1.4051\;CO_{2}+1.4051\;H_{2}$
	CCU	0.2452 CO_2_ released into the environment 1.8735 CO_2_ to the methanol CO_2_/H_2_ synthesis 0.3333 CO_2_ to the RWGS reactor
	CO_2_/H_2_ synthesis	$1.8735\;CO_{2}+5.6206\;H_{2}\to 1.8735\;CH_{3}OH+1.8735\;H_{2}O$
	SMR	$CH_{4}+H_{2}O\to CO+3H_{2}$
	Combustion	$0.5887\;CH_{4}+1.1774\;O_{2}\to 0.5887\;{CO}_{2}+1.1774\;H_{2}O$
	RWGS	$0.333\;CO_{2}+0.333\;H_{2}\to 0.333\;CO+0.333\;H_{2}O$
	CO/H_2_ synthesis	$1.3333\;CO+2.6666\;H_{2}\to 1.3333\;CH_{3}OH$
